# Alterations of Astrocytes in the Context of Schizophrenic Dementia

**DOI:** 10.3389/fphar.2019.01612

**Published:** 2020-02-07

**Authors:** Vadim V. Tarasov, Andrey A. Svistunov, Vladimir N. Chubarev, Susanna S. Sologova, Polina Mukhortova, Dmitrii Levushkin, Siva G. Somasundaram, Cecil E. Kirkland, Sergey O. Bachurin, Gjumrakch Aliev

**Affiliations:** ^1^I.M. Sechenov First Moscow State Medical University (Sechenov University), Moscow, Russia; ^2^Department of Biological Sciences, Salem University, Salem, WV, United States; ^3^Institute of Physiologically Active Compounds Russian Academy of Sciences, Chernogolovka, Russia; ^4^Federal State Budgetary Institution, Research Institute of Human Morphology, Russian Federation, Moscow, Russia; ^5^GALLY International Research Institute, San Antonio, TX, United States

**Keywords:** schizophrenia, astrocyte, N-methyl-d-aspartate, glutamate, glial fibrillary acidic protein, S100B, kynurenic acid

## Abstract

The levels of the astrocyte markers (GFAP, S100B) were increased unevenly in patients with schizophrenia. Reactive astrogliosis was found in approximately 70% of patients with schizophrenia. The astrocytes play a major role in etiology and pathogenesis of schizophrenia. Astrocytes produce the components that altered in schizophrenia extracellular matrix system which are involved in inflammation, functioning of interneurons, glio-, and neurotransmitter system, especially glutamate system. Astrocytes activate the interneurons through glutamate release and ATP. Decreased expression of astrocyte glutamate transporters was observed in patients with schizophrenia. Astrocytes influence on N-methyl-d-aspartate (NMDA) receptors *via* D-serine, an agonist of the glycine-binding site of NMDA receptors, and kynurenic acid, an endogenous antagonist. NMDA receptors, on its turn, control the impulses of dopamine neurons. Therefore following theories of schizophrenia are proposed. They are a) activation of astrocytes for neuroinflammation, b) glutamate and dopamine theory, as astrocyte products control the activity of NMDA receptors, which influence on the dopamine neurons.

## Introduction

Schizophrenia is a mental disorder, determined as a complex of positive, negative and cognitive symptoms (Carbon and Correll, 2014). Positive symptoms are the symptoms that present in patients with schizophrenia, but not healthy people, such as psychosis. Negative symptoms are the symptoms, associated with lack of functions, such as lack of motivation, reduction in spontaneous speech, and social withdrawal. Cognitive symptoms related to neurocognition: difficulties in memory, attention, and executive functioning (Van Os and Kapur, 2009). However, positive symptoms are more noticeable in patients than negative and cognitive symptoms, which helps in diagnosis of schizophrenia.

The dopamine theory of schizophrenia, based on hyperactive dopamine projections in the mesolimbic system and reduced dopamine projections in the mesocortical system (Juárez Olguín et al., 2016), is the prevalent explanation of schizophrenia symptoms now. Alterations in striatal D2 receptors cause positive symptoms, and impairments in the prefrontal cortex D1 receptors cause negative and cognitive symptoms (Lau et al., 2013). Traditional pharmacotherapy, based on the dopamine theory of schizophrenia, has several significant limitations. The use of antipsychotics improves predominantly positive symptoms, although there is evidence of improvement of negative symptoms with the use of clozapine (Kane, 1988) and aripiprazole (Veerman et al., 2017). Approximately 25% of patients are resistant to therapy (Remington et al., 2017), in addition, the rate of metabolic syndrome among patients was 32.5% (Mitchell et al., 2013), which worsens life quality and predisposes to cardiovascular diseases.

Glutamate theory of schizophrenia is based on the ability of N-methyl-d-aspartate (NMDA) antagonists, such as ketamine, induced schizophrenia-like psychosis (Steeds et al., 2015). Disturbances in NMDA receptors in interneurons lead to the absence of inhibition impulses to the glutamate neurons, increasing glutamate activity especially in the prefrontal cortex, which can be related to negative symptoms of schizophrenia. The agonists of metabotropic glutamate receptors mGluR2 demonstrate the antipsychotic activity in clinical trials (Moghaddam and Javitt, 2012).

Neuroinflammation theory of schizophrenia is based on increased expression of proinflammatory agents and the presence of autoantibodies. Epidemiological studies associate schizophrenia with autoimmune disorders, autoantibodies affect synapses and NMDA-type glutamate receptors and cause damages in the brain (Sawa and Sedlak, 2016). Inflammation processes, in its turn, are connected with oxidative stress—the imbalance between the production of reactive oxygen species radicals and antioxidant system. Interconnections of neurons and glia mediate the inflammation processes, that means that altered glial state will be an important point in schizophrenia research (Leza et al., 2015).

The glial theory of schizophrenia assumes that initial disturbances in glial cells can lead to the abnormalities of the neurons and neurotransmitters. The glial theory of schizophrenia based on the proven inflammatory response and elevated levels of the characteristic markers of active glia—S100B and glial fibrillary acidic protein (GFAP) (Wang et al., 2015). In schizophrenia patients there is an accumulation of the metabolite of tryptophan—kynurenic acid (KYNA) (Plitman et al., 2017), acting as an antagonist of NMDA receptors and altered glutamate transport—which binds glial and glutamate theory, as well as due to the influence on interneurons. Obviously, astrocytes as the most abundant glial cells, are the objects of careful attention at the researchers of a lot of central nervous system (CNS) diseases. But its role in the development of schizophrenia is insufficiently studied. The concept of our paper is the generalization of previously obtained data in this field. This review analyses the action of astrocyte both on schizophrenia symptoms and on the related with them factors, such as inflammation processes, extracellular matrix, and different neurotransmitters.

### Evidences of Alterations in Astrocyte System in Different Brain Areas

Patients with schizophrenia show increased activation of glia, especially astrocytes, which play a role in the development and functioning of synapses, glutamate release, water-electrolyte balance, regulation of blood circulation, and neuroprotection (Blanco-Suárez et al., 2017; Sullivan et al., 2018). The functions of astrocytes are related to the functions of other glial cells: protective functions of astrocytes can be changed by microglia, and also astrocytes, interacting with oligodendrocytes, play a role in myelination (Iglesias et al., 2017), which makes their role in schizophrenia even more significant.

The most part of *postmortem* human studies of astrocyte alterations in schizophrenia have focused on the number of glial cells. The number of astrocytes was reduced in the cingulate cortex (Williams et al., 2013a), motor cortex (Benes, 1986), medial, and ventrolateral regions of the nucleus accumbens (Pakkenberg, 1990), basal nuclei (Williams et al., 2013b), substantia nigra (Williams et al., 2014), but increased in the periventricular space (Bruton et al., 1990) and is not altered in the temporal and frontal cortex (van Kesteren et al., 2017), in the hippocampus (Schmitt et al., 2009), amygdala, and ventral pallidum in schizophrenia (Pakkenberg, 1990). The changes of the astrocyte density in the prefrontal cortex vary depending on the area of the dorsolateral prefrontal cortex of *postmortem* brain tissue (Rajkowska et al., 2002). Studies of the number of astrocytes in the mediodorsal nucleus of the thalamus vary: one study showed a decrease in the number of astrocytes (Pakkenberg, 1990), but another study showed increased GFAP expression in the mediodorsal nucleus of the thalamus and in the anteroventral, internal capsule, and putamen (Barley et al., 2009). A positive correlation has been found between the age of macaque monkey and the density of astrocytes in paralaminar nucleus (Chareyron et al., 2012) which suggests that different age of patients can contribute to the heterogeneity of astrocyte density.

Selemon et al. have found an increased density of glia in the prefrontal cortex in rhesus monkeys, chronically taking antipsychotics (Selemon et al., 1999). This is contradicted by the fact that the expression of clozapine- and haloperidol-induced Fos—protein in Sprague–Dawley rats is not colocalized with astrocytes, which suggests that haloperidol and clozapine do not act on these glial cells (Ma et al., 2003).

Individual astrocyte genes are associated with schizophrenia, which is proved by the increase in astrocyte Marker Gene Profile in the thalamic region in the transcriptomics analyses of *post-mortem* brain tissue (Toker et al., 2018). A significant number of changes in gene expression in schizophrenia patients occur in the anterior cingulate cortex, which is responsible for cognitive function, error recognition, and motivation, while very few or no significant expression differences in the dorsolateral prefrontal cortex and nucleus accumbens (Ramaker et al., 2017). Alterations in the expression of the two proteins are the most common among patients with schizophrenia—aldolase C (11 reports) and GFAP (9 reports), both expressed primarily by astrocytes (Davalieva et al., 2016). Adult astrocytes also express calcium-binding protein S100B, glutamate-aspartate transporter/excitatory amino acid transporter 1 (EAAT1), and glutamate transporter (GLT-1) (Iglesias et al., 2017). Markers of enhanced astrocyte response are usually GFAP and S100B (Kim et al., 2018; Michetti et al., 2019).

Glucose metabolism finishes with the formation of oxidative radicals, and astrocytes normally increase mobilization of glycogen and glucose utilization in the case of oxidative stress (Lavoie et al., 2011). Destruction of astrocyte lactate transporters produces a loss of memory, suggesting the importance of lactate transport in astrocytes for the formation of long-term memory in rats (Xia et al., 2016). Inhibition of glycogenolysis in rats impairs memory, but it is improved by the use of lactate, which can be related to the impairments in working memory in patients with schizophrenia (Newman et al., 2011).

#### Marker of Enhanced Astrocyte Response GFAP

GFAP is expressed by the astrocytes, perisinusoidal stellate cells of the liver, Leydig cells, glomeruli of the kidney, and chondrocytes of elastic cartilage (Buniatian et al., 1998). GFAP is a marker of reactive astrocytes, many astrocytes normally do not release detectable GFAP levels (Kim et al., 2018). GFAP expression is different in patients with schizophrenia (Catts et al., 2014). It was elevated in the anteroventral and mediodorsal thalamic nuclei and putamen (Barley et al., 2009), and in dorsolateral prefrontal cortex in patients with neuroinflammation (Catts et al., 2014). GFAP expression was significantly reduced in the in the frontal cortex and cingulate cortex of *postmortem* brain tissue (Williams et al., 2013b; Wang et al., 2015). The level of GFAP and the number of GFAP-positive cells were not statistically different in the hippocampal and neocortical regions (Pantazopoulos et al., 2010; Schnieder and Dwork, 2011). However, animal models of schizophrenia showed an increase in the level of GFAP (Kim et al., 2018). Variation in the data may be related to differences in age, causes of death, the severity of the inflammatory response, genetic characteristics, heterogeneity of schizophrenia symptoms (Schmitt et al., 2009; Schnieder and Dwork, 2011; Catts et al., 2014). The latter is also confirmed by the difference in astrocyte activation of transgenic mice by alpha-amino-3-hydroxy-5-methyl-4-isoxazolepropionic acid receptor (AMPAR) modulators (Höft et al., 2014). Moreover, multiple injections of the NMDA receptor antagonist that causes schizophrenia-like symptoms has led to increase in the level of GFAP in the hippocampus of rats (Yu et al., 2015) and in the medial prefrontal cortex of mice (Gomes et al., 2015).

#### Marker of Enhanced Astrocyte S100B

S100B is a calcium-binding protein, a biomarker of brain damage and stress that is synthesized by oligodendrocytes and astrocytes. It is also secreted by CD8T-lymphocytes and NK-cells during their stimulation, inducing microglial migration through increased cytokine expression (Michetti et al., 2019). The level of S100B was increased by almost two times compared to the healthy control groups, but its increase was observed in schizophrenia patients is not uniform (Aleksovska et al., 2014). The level of S100B was decreased in the *postmortem* brain tissue of deep layers of the anterior cingulate gyrus (Katsel et al., 2011) and in the corpus callosum (Steiner et al., 2014b). In the animal models, the level of S100B were heterogeneous (de Souza et al., 2015) in the hippocampus. S100B-immunopositive glia was elevated in paranoid as compared to residual schizophrenia patients (Steiner et al., 2008). There is evidence that S100B can be released by astrocytes in response to activation of 5-HT receptor, which means that an excess of serotonin can provoke the release of S100B, but this is opposed by serotonin and norepinephrine reuptake inhibitor in the hippocampus of rats (Michetti et al., 2019). Increased S100B expression can lead to metabolic disturbances in astrocytes and neurons, for example, reduced glucose uptake by astrocytes. The level of S100B in serum correlates with the development of insulin resistance in patients with schizophrenia (Steiner et al., 2014a). Intracellular S100B provokes proliferation, extracellular S100B provokes cell differentiation in small doses and induces cell death in large ones (Aleksovska et al., 2014). It was noted that antipsychotics (haloperidol and clozapine) reduce the level of S100B in the cell cultures (Steiner et al., 2010). The level of S100B in serum positively correlates with the manifestation of negative symptoms before treatment, while negative symptoms may be predictors of increased S100B. The level of S100B does not change during 6 months of treatment and the level of S100B also kept high after 6 months of treatment in patients with high rates of negative symptoms. Patients with increased S100B had problems with expression of emotions, communication with others, initiative, while mice with increased S100B showed impaired memory and learning ability (Rothermundt et al., 2004). Mice with S100B deletion had better fear memory in the contextual fear conditioning (Nishiyama et al., 2002).

#### Morphology of Astrocytes in Schizophrenia

Data about the presence of changes in the morphology of astrocytes in schizophrenia is also different: despite the hypertrophy of glial fibrillary acidic protein-containing cellular processes, the volume of tissue accessed by individual astrocytes of mice remains unchanged (Wilhelmsson et al., 2006). Reactive astrogliosis was found in approximately 70% of patients with schizophrenia in the thalamus, limbic system, and periventricular space (Mallya and Deutch, 2018), although reactive astrogliosis was not found in the entorhinal cortex (Casanova et al., 1990; Schnieder and Dwork, 2011). Gliosis at the rostral and caudal ends was more common in patients with late onset of schizophrenia (Nasrallah et al., 1983; Schnieder and Dwork, 2011). Differences in the data can be partly explained by differences in the age of the patients (Schmitt et al., 2009). The morphology of astrocytes depends on the measure of the inflammatory response in patients with schizophrenia, so the differences in morphology can partially explain the different data about the levels of GFAP (Kim et al., 2018). Morphological changes of astrocytes can also alter neuronal networks, which can apparently contribute to the development of schizophrenia symptoms (Poskanzer and Yuste, 2011). The appearance of astrogliosis can be expected due to the continuous patients neurodegenerative state, which can be predicted by altering the volume of brain areas (Brugger and Howes, 2017), but the data on its manifestation is different, which can be explained by the presence of different types of astrogliosis and the short duration or blockade of astrocyte reactions in many people with schizophrenia (Catts et al., 2014). Normal astrogliosis has provided several significant benefits, including protection of neurons, recovery of the blood-brain barrier, and reducing inflammation in the central nervous system, while small and medium astrogliosis can look the same with healthy astrocytes of the CNS (Sofroniew, 2015). Pathological astrogliosis can lead to harmful effects: to provoke and increase inflammation, to produce molecules that destroy the blood-brain barrier and facilitate the migration of leukocytes into the CNS parenchyma (Leza et al., 2015; Sofroniew, 2015). The relationship between taking atypical antipsychotics and the suppression of astrogliosis, (including that caused by NMDA antagonist) have been also discovered (Catts et al., 2014).

Reactive hypertrophic astrocytes lose spontaneous Ca^2+^ oscillations *in situ* after stab wound injury, controlling the emission of gliotransmitters, which may be related to the neural network (Rossi, 2015). There is evidence that the release of high concentrations of tumor necrosis factor (TNF)-α by reactive microglia shows Ca^2+^-dependent release of gliotransmitter glutamate by astrocytes, leading to neuronal damage (Bezzi et al., 2001).

Myoinositol is a marker of glial activation, for which the connection with astrocytes for schizophrenia spectrum disorders patients has been found (Chiappelli et al., 2015). Its level may be associated with inflammation and increased in untreated individuals. The level of myoinositol in the striatum positively correlates with the level of glutamate in untreated patients and positive symptoms, but not correlated with negative or symptoms at all (Plitman et al., 2016).

#### Prenatal Infection and Mother Deprivation Lead to Schizophrenia

Prenatal infection is one of the suspected causes of schizophrenia and it can make astrocytes hypersensitive to stimuli in the future, which may cause an enhanced response in the central nervous system (Takahashi and Sakurai, 2013). Maternal immune activation on day 12 of mice embryonic development leads to changes in astrocytes and microglia and increases the GFAP levels, which indicate astrogliosis in the amygdala (O'Loughlin et al., 2017), but in another study, mice prenatal immune activation did not change astrocyte density (Giovanoli et al., 2016). Activation of astrocytes during rat embryonic development can disrupt the cortical and thalamocortical formation (Beamer et al., 2017).

Mother deprivation in rats leads to specific behavioral symptoms of schizophrenia, which may be associated with corticosteroids. Mother deprivation provokes a higher level of GFAP and a large number of GFAP-positive astrocytes, which may indicate reactive gliosis (Llorente et al., 2009). At the same time, it has been found that mother deprivation provokes an increase in GFAP-positive cells only in male rats (López-Gallardo et al., 2008).

### Contribution of Astrocytes in Neuron-Neuron and Neuron-Glia Interaction

#### Inflammatory Processes and Astrocytes

**Figure 1:**
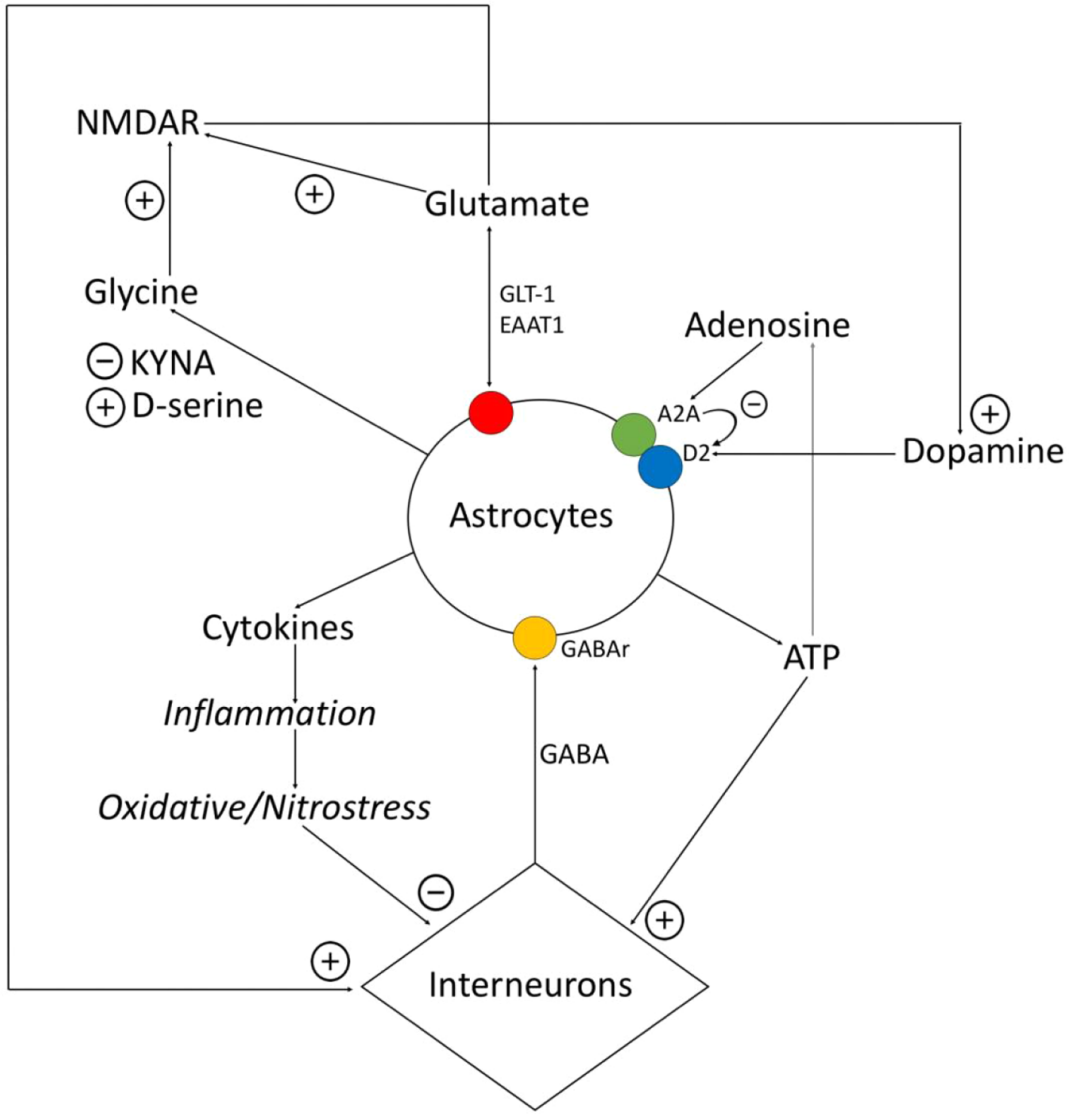
Relationship between astrocytes, interneurons, and transmitters. Astrocytes express proinflammatory cytokines, which provokes the destruction of gamma-aminobutyric acid (GABA)ergic interneurons. GABA influences on astrocytes and *via* Ca^2+^-dependent stimulates expression of different gliotransmitters (glutamate, ATP, cytokines). Glutamate and ATP have an activating impact on interneurons. Adenosine operates on A2A receptors of astrocytes, related with the dopamine receptors, and inhibits them. Astrocytes control the amount of glutamate *via* glutamate transporter (GLT)-1, excitatory amino acid transporter (EAAT)1, and also metabotropic glutamate receptors (mGluRs). Astrocytes express D-serine and kynurenic acid (KYNA), while D-serine is the agonist of N-methyl-d-aspartate (NMDA)-receptors, and KYNA is the antagonists of glycine site of NMDA-receptors. NMDA-receptors activate dopamine neurons.

Oxidative and nitrostress are the main mechanisms by which inflammation can generate cell damage (Leza et al., 2015). Hypotheses of oxidative stress as a cause of schizophrenia suggest that inflammation can act in the embryonic period of development and induce oxidative stress in fetal cells through cytokines that cross the placenta; or an inflammatory agent, such as an infection that causes an immune response and oxidative stress, can act on the brain in adolescence, causing characteristic changes (**Figure 1**). At the same time, oxidative and nitro stress can be caused by non-inflammatory stimuli (mitochondrial dysfunction, dopamine, hyperhomocysteinemia, smoking, hypofunction of NMDA receptors, etc.) (Leza et al., 2015; Nedic Erjavec et al., 2018). Oxidative stress is observed in the prefrontal cortex, anterior cingulate cortex and hippocampus, which matches with the places of altered astrocyte activity in schizophrenia. Oxidative stress inhibits the activity of NMDA receptors, which can lead to the appearance of ketamine-like symptoms, and has inhibitory effects on glycogen metabolism in mice (Lavoie et al., 2011). In this case, free radicals can cross the blood-brain barrier, which becomes more permeable due to the product of lipid peroxidation 4-hydroxynonenal (Nedic Erjavec et al., 2018) and other agents, causing an inflammatory response and a decrease in pH as a result of chronic stress (van Kesteren et al., 2017). However, under the influence of certain circumstances astrocytes can emit molecules, such as sonic hedgehog, which provoke the repair of blood-brain barrier (Sofroniew, 2015)

Interneurons are very sensitive to damage from oxidative stress, especially at the beginning of postnatal development (Grace, 2016). Prolonged stress increases the activity of dopamine neurons through the ventral tegmental area and increases the level of dopamine in the prefrontal cortex and nucleus accumbens. The medial prefrontal cortex regulates the amygdala’s response to stress. Stress-induced hyperreactivity of the amygdala leads to the loss of parvalbumin interneurons (Grace, 2016) and changes in their proportions in the hippocampus, which leads to even greater hyperexcitation of dopamine systems, and this causes symptoms of schizophrenia (Grace, 2016).

The main cells of the immune response in the CNS are astrocytes and microglia. In this case, microglia mainly produces type 1 cytokines, such as interleukin (IL)-12, and astrocytes inhibit the production of IL-12 and produce type 2 cytokines, for example, IL-10 (van Kesteren et al., 2017). The dysregulation of this balance can damage the neurons, cause a deficit of interneurons, leading to alteration of oligodendrocytes and inhibition of gamma-aminobutyric acid (GABA) interneurons (Nedic Erjavec et al., 2018). IL-1β and IL-6 promoted dopaminergic transmission (Purves-Tyson et al., 2019).

Stimulation of mGluR5 and a1-noradrenergic astrocyte receptors provokes mild inflammatory processes, including the release of prostaglandins and other eicosanoids, which can regulate communication between neurons and blood vessels (Rossi, 2015).

Experimental and clinical observations have shown that loss or dysfunction of astrocytes can seriously exacerbate CNS inflammation and tissue damage. Normal astrocytes produce pro-inflammatory agents, such as cytokine Il-6, IL-10, IL-17, and IL-1ß that attract leukocytes through vasodilation. Then the astrocytes exhibit a modulatory role to form the necessary barriers to limit the inflammation or enhancing the anti-inflammatory process through vasoconstriction mechanism. Hence astrocyte transcriptome changes that are shifted by pathogen-associated molecular patterns (PAMPSs) including LPS and associated cytokines (Sofroniew, 2015). Astrocytes also regulate the function of microglia during injury or recovery of the brain *via* secreted cytokines (Miyake et al., 2011; Bouzier-Sore and Pellerin,2013; Bernstein et al., 2015; Andrade 2016; Dean et al., 2016).

#### The Role of Astrocytes in the Functioning of the Extracellular Matrix

During pathogenesis of schizophrenia, glia loses the ability to form compartments and connections, which leads to disruption of perception and an inability to think, which can be included in the development of cognitive symptoms in schizophrenia. Supposing that astrocyte gap junctions are the site of memory formation and intentional programming, their functions must be essential for cognition and higher information processing (Mitterauer, 2011). This suggests changes in extracellular matrix system, plays a role in the pathogenesis of schizophrenia (Berretta, 2012; Takahashi and Sakurai, 2013). The extracellular matrix is synthesized by neuronal and glial cells. In humans, unlike other mammals, there is a large number of astrocytes, synthesizing chondroitin sulfates as extracellular matrix in the amygdala. The patients with schizophrenia showed a large increase in chondroitin sulfate proteoglycan (CSPG) - positive glial cells in the deep amygdala and entorhinal cortex and the density of GFAP - positive cells was not changed at some studies (Pantazopoulos et al., 2010). CSPGs play a role in adult synaptic plasticity (Chelini et al., 2018). The extracellular matrix influences synapses’ stabilization and maturation in different ways. Firstly, the rate of viscosity of the matrix and the interaction between the negatively charged chains of the glycosaminoglycan-proteoglycan and glutamate controls the diffusion of neurotransmitter in the extracellular space (Deutsch et al., 2010). Secondly, the extracellular matrix at the level of hyaluronan separates the surface of neurons, limiting the surface mobility of integral membrane proteins, including glutamate receptors in rats (Frischknecht et al., 2009). Also, the altered composition of extracellular matrix can lead to excessive diffusion of dopamine into the extracellular space from excessive stimulation extrasynaptic D2 receptors.

Reduced expression of Reelin, which is also a part of extracellular matrix, was noted in patients with schizophrenia in several brain regions, including the hippocampus, prefrontal and temporal cortex, cerebellum and caudate nucleus (Eastwood and Harrison, 2006; Guidotti et al., 2011). In adulthood, Reelin is expressed mainly by GABAergic interneurons (Dong et al., 2007). Reduction of Reelin regulation is usually accompanied by a decrease in glutamic acid decarboxylase expression, indicating a strong functional relationship between Reelin expression and GABAergic neurotransmission (Eastwood and Harrison, 2006). Therefore, the changes in extracellular matrix have an influence on release of neurotransmitters both directly and *via* inhibitory GABAergic interneurons.

### Astrocyte-Related Changes in Transmitters Systems

#### Glutamate System

In schizophrenia, there is hypofunction of NMDA receptors, which leads to a decrease in prefrontal cortex functions (Herédi et al., 2017). Weakened prefrontal cortex function associated with NMDA receptor hypofunction may be involved in the development of negative and cognitive symptoms. This is confirmed by the detection of anti-NMDA antibodies in patients with the first episode of schizophrenia (Levite, 2014). The use of antagonists of NMDA receptors phencyclidine and ketamine, cause the psychotic reactions and leads to hyperactivation of dopamine (Rial et al., 2014). NMDA receptors are involved in synaptic plasticity, which plays a role in learning and memory (Parpura and Verkhratsky, 2012). MK-801 is an NMDA antagonist, increasing the number of GFAP-positive astrocytes in the prefrontal cortex (Gomes et al., 2015). Clozapine reduces the manifestations of these changes, however, an indirect connection through the dysfunction of GABAergic interneurons is possible. Also, an increase in the level of astrocytes can be compensation in response to a decrease in the level of glutamate.

The basis of the modified glutamate transmission is increased glutamate excretion in the hippocampus, in which the dysfunction of inhibitory interneurons in the hippocampus and prefrontal cortex also plays a role (Millan et al., 2016). Reduced activation of NMDA on inhibitory interneurons leads to increased release of glutamate by pyramidal hippocampal neurons (Tayeb et al., 2019).

The reason for the decrease in the activity of parvalbumin neurons in schizophrenia patients in the reduced access to glutamate (Chung et al., 2016). *Postmortem* studies of patients with schizophrenia showed a simultaneous decrease in the levels of parvalbumin interneurons (caused by changes in the expression of parvalbumin) and glutamic acid decarboxylase in the dorsolateral prefrontal cortex (Toker et al., 2018). In this case, NMDA antagonists cause a decrease in the level of mice parvalbumin neurons in the prefrontal cortex, but not in the hippocampus (Gomes et al., 2015). Astrocytes transformer at the activity of the inhibitory GABA from interneurons to excitatory glutamate activity increases synaptic transmission. Astrocytes also induce the increase of inhibitory synaptic connections of interneurons through glutamate release and activation kainate receptors in the inhibitory terminals (Perea et al., 2016), ATP secreted by astrocytes, has also an activating effect on mice interneurons (Bowser, 2004).

Ketamine, NMDA antagonist, decreases Ca2+ transients in astrocyte cell culture, which affects the secretory activity of astrocytes (Lasič et al., 2019). In this case, ketamine does not regulate exocytosis directly through cAMP. Ketamine-induced increase in the density of cholesterol domains in astroglial plasmalemma may stimulate the release of cholesterol molecules by astrocytes to neurons, which may be critical for the morphological plasticity of synapses. Structural changes in astroglial plasmalemma likely involve adenylate cyclase, which increases cAMP in the absence of stimulation of G-protein-coupled receptor (**Figure 1**).

Glutamate transporters GLT-1 and EAAT1 are localized in astrocytes and are responsible for glutamate uptake in astrocyte (Rial et al., 2014). Astrocytes are also responsible for the conversion of glutamate to glutamine, and changes in the glutamate/glutamine cycle that impact the energy exchange between neurons and astrocytes to cause schizophrenia (Sullivan et al., 2018).

In schizophrenia, there is a decreased expression of GLT-1 in the hippocampus of *postmortem* brain samples (Shan et al., 2013) and prefrontal cortex of genotyped patients (Spangaro et al., 2012). The blockade of GLT-1 increased the tonic activation of presynaptic metabotropic glutamate receptors (mGluRs) (Fleming et al., 2011), some of which protect neurons from excessive excitability and regulate the functioning of working memory of rhesus macaques (Jin et al., 2018). It was also noted that agonist mGluR2/3 ameliorates symptoms of schizophrenic psychoses (Segnitz et al., 2009).

In schizophrenia, there is also a decrease in the expression of EAAT1 in the prefrontal cortex. Mice with EAAT1 deficiency showed schizophrenia-like behavior and were more sensitive to locomotor hyperactivity caused by NMDA antagonists. Thus, the locomotor hyperactivity caused by the lack of EAAT1 was reduced haloperidol. Mice with reduced EAAT1 levels also showed cognitive symptoms. The lack of EAAT1 makes the cells more sensitive to various traumatic factors (Mei et al., 2018).

Haloperidol and clozapine reduce GLT-1 and EAAT3 levels in rodents, while aripiprazole reduces EAAT1 expression but has minimal effect on GLT-1, which may further lead to differences in effects on positive, negative, and cognitive symptoms (Segnitz et al., 2009; Mei et al., 2018).

#### Glycine System

Glycine is an NMDA receptor agonist that can be released from astrocytes through activation of glutamatergic non-NMDA-type ionotropic receptors (Harsing and Matyus, 2013). In this case, the synaptic form of NMDA has a low affinity for glycine, and non-synaptic NMDA—high (Balu, 2016). Inhibitors of non-synaptic GlyT-1, which lead to an increase in the non-synaptic glycine concentration in rats (Harsing and Matyus, 2013), may participate in the actions of drugs on patients, including the previously described antipsychotic effect (Tsai et al., 2004) in positive, negative, and cognitive symptoms. D-serine, produced by astrocytes, is an agonist of the glycine-binding site of NMDA receptors (Möller and Czobor, 2015). The association of serine racemase, synthesizing serine enzyme, with schizophrenia was found, as well as mice with a lack of serine racemase gene showed behavior similar to schizophrenia (Takahashi and Sakurai, 2013).

#### Kynurenic Acid as N-Methyl-D-Aspartate Antagonist

Tryptophan is elevated in the cerebrospinal fluid of patients with schizophrenia, along with one of its metabolites—kynurenic acid (KYNA) (Linderholm et al., 2012; Kegel et al., 2017; Tayeb et al., 2019). Ninety percent of tryptophan is metabolized in KYNA. In schizophrenia discovered the lack of kynureninase in astrocytes that can be one of the reasons for increasing the level of KYNA in schizophrenia (Plitman et al., 2017). The conversion of kynuren to KYNA takes place primarily within astrocytes, as these cells contain KATs and therefore cannot degrade kynuren to its metabolites. Of the four existing KATs, KAT II is thought to be the main enzyme of KYNA production.

KYNA acts as an antagonist of all 3 ionotropic glutamate receptors, including NMDA, AMPA, and receptors kainate, while KYNA is the only currently known endogenous antagonist of NMDA (Plitman et al., 2017). KYNA presumably acts as an endogenous antagonist of the glycine site of the NMDA receptor and as a negative allosteric regulator of the nicotinic acetylcholine (nACh)-receptor α7 (Kozak et al., 2014). Both NMDAR and nACh-receptor α7 contribute to the functioning of working memory, and elevated levels of KYNA may contribute to NMDA-hypofunction, cognitive deficits, and negative symptoms. In high micromolar concentrations in rats KYNA acts as an NMDA antagonist, and in lower concentrations reduces the excitability of neurons through mechanisms independent of NMDA (Alkondon et al., 2011).

Conversion kynurenine to KYNA occurs mainly in astrocytes since these cells contain kynurenine aminotransferase (KAT) (Plitman et al., 2017). In adult mouse brain KAT-2 is expressed not only by astrocytes but also by neurons in several brain regions (hippocampus, substantia nigra, striatum, and prefrontal cortex), while the structure of the brain consisting mainly of GABAergic neurons (e.g., the substantia nigra), have the strongest neuronal expression of KAT-2 (Herédi et al., 2017). In rats KAT-2 inhibition reduces KYNA levels and improves cognitive function (Kozak et al., 2014). Activation of astrocytes can increase the production of KYNA (Plitman et al., 2017). The introduction of IL-6 and an increase of prostaglandin E2 level in cultured human astrocytes increases KYNA. Atrophic astrocytes also showed increased production of KYNA.

The possible role of KYNA as a functional link between the stimulation of dopamine receptors and the neurotoxicity of NMDA in the striatum was noted in rats (Poeggeler et al., 2007). An increase in KYNA leads to a decrease in the levels of dopamine, acetylcholine, GABA, and glutamate (Plitman et al., 2017). These inverse associations remain unclear in schizophrenia, as typical neurotransmitter disorders, such as increased synthesis and release of dopamine in the striatum, and elevated levels of subcortical glutamate, seem incompatible with the observed increase in KYNA levels. Antipsychotics normalize the level of tryptophan and reduce the production of KYNA (Müller et al., 2014). Pharmacologically important targets are the enzymes kynurenic way, and also cyclooxygenase-2, which reduces the level of KYNA.

The impact of the increased level of KYNA in schizophrenia symptoms is ambiguous. Elevated levels of KYNA provoked cognitive defects in animals: auditory sensory gating, prepulse inhibition, contextual discriminations, spatial working memory (Alexander et al., 2012). The level of KYNA in cerebrospinal fluid positively correlates with overall psychotic symptoms positive and negative symptoms. The symptom score included results from the scales measuring positive and negative psychotic symptoms (SAPS and SANS), the scale for schizotypal personality traits (SPQ-B), and the interview for schizoid, schizotypal, or paranoid personality traits (SCID-II interview cluster A section) (Kegel et al., 2017). In animal models, increased KYNA is associated with cognitive deficits, including deficits in spatial and working memory (Kozak et al., 2014). The higher initial level of KYNA in plasma was associated with a greater reduction in positive symptoms on the Positive and Negative Syndrome Scale as a result of therapy (Plitman et al., 2017). Thus, preclinical studies have demonstrated the effect of kynureninase acid as the behavior (e.g., cognitive function) and neurotransmission (e.g., glutamatergic, dopaminergic).

#### Gamma-Aminobutyric Acid System

GABA acts on astrocytes through GABA receptors, contributing to the release of chlorine and depolarization of astrocytes, and GABA receptors, activating calcium-dependent mechanisms and contributing to the growth of gliotransmitter (glutamate, ATP, cytokines) (Losi et al., 2014; Rossi, 2015). Activation of presynaptic GABA receptors increases the inhibitory effects of interneurons. At the same time, the activation of GABAb receptors leads to the activation of mGlu receptors of types 2 and 3, which leads to synaptic depression (Perea et al., 2016). There is evidence that mGlu2/3 receptor agonists can be used as atypical antipsychotics (Aghajanian, 2009).

GABA entering the astrocyte is mediated by a GABA-transporter operating on the principle of symport with sodium, increasing the content of intracellular sodium can contribute to the reversible operation of GABA-transporter (Losi et al., 2014). Reduced GABA release by reactive astrocytes may be important in reducing hippocampal synaptic plasticity, learning, and memory in mice (Rossi, 2015). The blockade of astrocyte GABA receptors improves cognitive abilities, and their complete removal destroys the ability to learn.

#### Adenosine System

Adenosine acts through two types of receptors—A1 and A2 (Rial et al., 2014). A1-receptors inhibit the release of neurotransmitters, including glutamate. Activating A2A-receptors increases the release of glutamate, supporting the activation of NMDA receptors and inhibits A1-receptors (Boison et al., 2012). At the same time, A2A receptors are not directly related to glutamate release mechanisms. A2A receptors are located in areas rich in dopamine: prefrontal cortex and striatum, and their activation leads to vasodilation and decreased dopaminergic innervation. The blockade of A2A receptors led to the delayed appearance of interneurons in the hippocampus and degradation of working memory (Kim et al., 2018). Activation of A1-receptors localized in oligodendrocytes stimulates myelination, and A2-receptors inhibit the proliferation of oligodendrocytes (Rial et al., 2014).

Recently gaining popularity adenosine theory of schizophrenia. It consists of the hyper-activation of adenosine kinase, which reduces the level of adenosine (Rial et al., 2014). It is shown that selective elimination of astrocyte A2A receptors in mice is related with deficits in GLT-1 activity (Matos et al., 2012; Kim et al., 2018). Also, ATP released by astrocytes is converted into adenosine, which inhibits the release of glutamate through presynaptic A1 receptors (Losi et al., 2014). Preclinical studies have shown that mice without adenosine A2A receptors in astrocytes demonstrate a potential response to the NMDA antagonist in the locomotor activity test (Kim et al., 2018).

Striatal astrocytes express the heterodimer native receptors A2A-D2 (Cervetto et al., 2017). D2 receptors inhibit presynaptic glutamate release, while A2A receptor activation eliminates the effect of D2 receptor activation (Aliagas et al., 2013). A study was carried out using a synthetic peptide VLRRRRRKRVN, corresponding to the receptor region involved in the electrostatic interaction between A2A and D2 receptors. It was shown that this peptide eliminated the ability of the A2A receptor to counteract the effect mediated by the D2 receptor (Azdad et al., 2009). Hypofunction of A2A receptors in the striatum can lead to hyperfunction of D2 receptors, which are involved in disorders associated with neuroinflammatory processes, stimulating immune responses and increasing the resistance of dopaminergic neurons to neurotoxic damage. Dysfunction striatal astrocytic A2A receptor, mediated damage of the D2 receptor, break down the homeostasis of glutamate and was presumably associated with schizophrenia (Ciruela et al., 2006).

#### Dopamine System

The morphological basis of the accepted dopaminergic theory of schizophrenia is the dysregulation of the dopaminergic system primarily in the striatum (Chuhma et al., 2017; Weinstein et al., 2017), which includes not only an excess of stimulation of dopaminergic neurons but also a violation of their communication and activity (Laruelle, 2014).

*Postmortem* examinations showed an increase in the level of dopamine in the striatum and an increase in the density of the D2 receptor but without changes in dopamine active transporter (DAT) densities (Howes et al., 2015). Interestingly, in individuals who do not receive antipsychotics, the density of D2 receptors has not been increased, unlike those treated with antipsychotics. Most likely, this is because all currently licensed antipsychotics bind to D2 and D3 receptors.

In schizophrenia, there was a decrease in the number of synapses in the striatum, which controls the lateral ventral part of the tegmental area and the black substance (Grace, 2016). Impulses of dopamine neurons in the ventral sides are controlled through NMDA receptors. Only in already activated NMDA receptors, dopamine neurons can emit neurotransmitters (Azdad et al., 2009).

Dopaminomimetic drugs, including amphetamine, in rodents provoke an increased release of dopamine from the striatum and induce positive symptoms similar to acute paranoid psychosis (Peleg-Raibstein et al., 2008; Rial et al., 2014). Injections of amphetamine in rats do not destroy cells or induce gliosis, as evidenced by the absence of an increase in the level of GFAP in dorsal caudate-putamen (Peleg-Raibstein et al., 2008). With increasing levels of dopamine in the cerebral cortex of rats in astrocytes, the co-localization of NMDA with GFAP significantly decreased (Ding et al., 2014).

Also, in schizophrenia, there was a lack of dopaminergic stimulation of the prefrontal cortex. This may be the result of impaired communication between the striatum and the prefrontal cortex, including a violation of NMDA receptors, a reduced level of which in schizophrenia is noted both in the prefrontal cortex and in the striatum in *postmortem* brain study (Errico et al., 2013).

## Conclusion

As a result of our study, we can conclude that astrocytes allow us to look at the etiology and pathogenesis of schizophrenia from a new point of view. They can explain the disparate data on morphological, metabolic and transmitter changes in the brain in schizophrenia. Astrocytes perform a supporting function for neurons, which is reflected in their ability to influence the concentration of transmitters both inside and outside the synaptic gap. Astrocytes mold the effects of dopamine in the striatal and cortical pathways through the release of glutamate and its effect on NMDA receptors. Other different mediators (adenosine, GABA, glycine) also take part in it. The presence of markers of activation of glia (S100B, GFAP, myoinositol) in serum and cerebrospinal fluid indicates a growing activation of astrocytes. Isolation of pro-inflammatory agents (cytokines, interleukins, and chemokines) and KYNA indicates a violation in the metabolism of astrocytes and surrounding cells. As a result, this leads to changes in the structure of the brain. Morphological manifestations include a decrease in astrocyte density in the frontal cortex, changes in the composition of the extracellular matrix and glial hypertrophy. Of course, in addition to the obvious changes in the brain, astrocytes make a significant contribution to the negative, positive, and cognitive symptoms of schizophrenia.

The number of astrocytes was reduced in the prefrontal cortex, that connects altered astrocyte system with mesocortical system, and nucleus accumbens, anterior cingulate cortex, which proves the changes in glial cells in the mesolimbic system, although in the hippocampus it was increased. The differences between the number of astrocytes in the mesocortical and mesolimbic system can affect on the manifestation of schizophrenia symptoms. Associations between GFAP and symptoms have not been found. At the same time the levels of another astrocyte markers, S100B and myoinositol, positively correlated with negative and positive symptoms, respectively. This finding suggests the dual alterations in astrocyte in brain regions, related with different symptom complexes.

The imbalance between microglia and astrocytes, which occurs in neuroinflammation, influence on different neurotransmitters, such as GABA and dopamine. GABAergic interneurons, affected by oxidative stress, modulate the activity of prefrontal cortex, hippocampus and amygdala, that worsen alterations in dopamine system and, therefore, symptoms of schizophrenia.

The contribution of glia to the development of cognitive symptoms was unexpected; normally it forms compartments and connections between neurons, but altered astrocyte themselves and extracellular matrix, affected by them, disturbs these interconnections.

NMDA receptors, related with all the groups of schizophrenia symptoms, associated also with astrocytes, since NMDA antagonist increased the number of GFAP-positive astrocytes in the prefrontal cortex. Astrocyte affects as on the glutamate system *via* KYNA and altered in schizophrenia glutamate transporters, as on the glycine system *via* non-synaptic GlyT-1 and D-serine. NMDA receptors and adenosine receptors, on its turn, control the dopamine release which is still considered the main schizophrenia neurotransmitter.

The study of the contribution of astrocytes to the etiology, pathogenesis, and symptoms of schizophrenia is associated with certain problems. The researches have been focused on the study of glia in different areas of the brain, which not only makes it difficult to generalize and analyze heterogeneous reactions of astrocytes but also eliminates the relationship between these areas and their respective astrocytes. In particular, the glia of “striatum-prefrontal cortex” axis, which supposedly plays a major role in the pathogenesis of schizophrenia, requires further analysis to study the contribution of NMDA receptors. Also, the study of the genetic patterns of astrocyte pathology is needed. Some problems are associated with the astrocytes themselves, for example, there is evidence of their heterogeneity, which means that it is impossible to accurately judge the suppression or activation of astrocytes in any structure of the brain. This problem is supplemented by the dependence of astrocyte functioning on the age of patients, which is not always taken into account in case-control studies.

Further study of the effect of astrocytes on neurotransmission may clarify the currently controversial aspects of brain function in schizophrenia and explain the characteristic symptoms. For example, it is not clear why a decrease in the expression of NMDA receptors is observed in both the prefrontal cortex and the striatum, if NMDA receptors have an activating effect on dopamine neurons. A separate role in this can play KYNA, NMDA receptor antagonist, an association of which was found with all types of symptoms of schizophrenia. Special attention should be paid to the study of GLT-1, whose effect on the symptoms of schizophrenia is heterogeneous (**Table 1**). The increased level of astrocyte activation markers in many areas of the brain indicates the need for further study of the theory of inflammation in schizophrenia in general and astroglial/microglial balance in particular. Finally, it is impossible to ignore data on changes in the metabolism of neurons and glia in schizophrenia, which can also contribute to the manifestation of the disease (**Table 1**).

**Table 1:** Association between symptoms of schizophrenia and metabolites, receptors, and pharmacological agents related with astrocytes.

Positive symptoms	Negative symptoms	Cognitive symptoms
Myoinositol in striatum ↑ (Plitman et al., 2016) Kynourenic acid ↑ (Kegel et al., 2017) EAAT1 ↓ (Mei et al., 2018)	S100B in serum ↑ (Rothermundt et al., 2004) Kynourenic acid ↑ (Kozak et al., 2014; Kegel et al., 2017)	S100B in serum ↑ (Nishiyama et al., 2002; Rothermundt et al., 2004) Kynourenic acid ↑ (Kozak et al., 2014) Destruction of lactate transporters ↑ (Xia et al., 2016) EAAT1 ↓ (Mei et al., 2018) A_2_A receptors ↓ (Kim et al., 2018)
Agonists mGluR2/3 ↓ (Segnitz et al., 2009) Inhibitors GlyT1 ↓ (Tsai et al., 2004)	Inhibitors GlyT1 ↓ (Tsai et al., 2004)	Inhibitors GlyT1 ↓ (Tsai et al., 2004) Inhibitors KAT-2 ↓ (Kozak et al., 2014)

↑—positive association, magnification of symptoms; ↓—negative association, diminution of symptoms.

## Author Contributions

VVT, AAS, VNC, SSS, PM, DL, SGS, CEK, SOB, and GA conceptualized and designed the study. PM and DL collected and analyzed the data. All of the authors discussed the analyses, the results, and their interpretation, revised and improved the various drafts. VT, AAS, VNC, SSS, PM, DL, SGS, CEK, SOB, and GA wrote the original draft. All authors have reviewed and approved the manuscript before submission.

## Funding

This research was supported within the framework of the grant provided by CSP Ministry of the Health Russian Federation, and by the IPAC RAS State Targets Project # 0090-2019-0005”. This work was also supported by the Russian Academic Excellence Project “5-100” for the Sechenov University, Moscow, Russia.

## Conflict of Interest

GA is employed by GALLY International Biomedical Research LLC, San Antonio, TX, USA.

The remaining authors declare that the research was conducted in the absence of any commercial or financial relationships that could be construed as a potential conflict of interest.
